# Crystal structure of the NS3-like helicase from Alongshan virus

**DOI:** 10.1107/S2052252520003632

**Published:** 2020-04-10

**Authors:** Xiaopan Gao, Kaixiang Zhu, Justyna Aleksandra Wojdyla, Pu Chen, Bo Qin, Ziheng Li, Meitian Wang, Sheng Cui

**Affiliations:** a NHC Key Laboratory of Systems Biology of Pathogens, Institute of Pathogen Biology and Center for Tuberculosis Research, Chinese Academy of Medical Sciences and Peking Union Medical College, Beijing 100730, People’s Republic of China; b Swiss Light Source at the Paul Scherrer Institute, CH-5232 Villigen, Switzerland

**Keywords:** Alongshan virus, NS3-like helicase, *Flavivirus*, segmented viruses

## Abstract

Alongshan virus (ALSV) is an emerging human pathogen that was identified in China and rapidly spread to the European continent in 2019, raising concerns about public health. ALSV belongs to the distinct *Jingmenvirus* group within the *Flaviviridae* family with segmented RNA genomes. While segments 2 and 4 of the ALSV genome encode the VP1–VP3 proteins of unknown origin, segments 1 and 3 encode the NS2b–NS3 and NS5 proteins, which are related to *Flavivirus* nonstructural proteins, suggesting an evolutionary link between segmented and unsegmented viruses within the *Flaviviridae* family. Here, the enzymatic activity of the ALSV NS3-like helicase (NS3-Hel) was characterized and its crystal structure was determined to 2.9 Å resolution. ALSV NS3-Hel exhibits an ATPase activity that is comparable to those measured for *Flavivirus* NS3 helicases. The structure of ALSV NS3-Hel exhibits an overall fold similar to those of *Flavivirus* NS3 helicases. Despite the limited amino-acid sequence identity between ALSV NS3-Hel and *Flavivirus* NS3 helicases, structural features at the ATPase active site and the RNA-binding groove remain conserved in ALSV NS3-Hel. These findings provide a structural framework for drug design and suggest the possibility of developing a broad-spectrum antiviral drug against both *Flavivirus* and *Jingmenvirus*.

## Abstract 2

The structural characterization of the NS3-like helicase of a segmented virus from the *Flaviviridae* is reported.

## 1. Introduction   

Alongshan virus (ALSV) is a novel tick-borne virus associated with human disease that was first identified in northeastern China (Wang *et al.*, 2019 ▸). Patients infected with ALSV present with fever, persistent headache, fatigue and nausea. Most confirmed ALSV cases had a clear history of tick bites. Soon after the identification of ALSV, the RNA of the virus was detected in *Ixodes ricinus* ticks in Finland; these ticks are a common species across Europe (Kuivanen *et al.*, 2019 ▸). ALSV is a positive-sense single-stranded RNA virus with a segmented genome. It was classified into the *Jingmenvirus* group of the *Flaviviridae*. Another segmented tick-borne virus capable of infecting humans is Jingmen tick virus (JMTV). JMTV was first identified approximately a decade ago in *Rhipicephalus microplus* ticks in China (Jia *et al.*, 2019 ▸). The emergence and spread of these novel pathogens pose potential threats to human health and an in-depth study of the proteins encoded by these viruses is thus warranted.

Most viruses of the *Flaviviridae* family have an unsegmented genome. These include the *Flavivirus*, *Pestivirus*, *Hepacivirus* and *Pegivirus* groups (Qin *et al.*, 2014 ▸). They host an ∼11 kb positive-sense single-stranded RNA genome containing a single open reading frame that encodes a polyprotein precursor. The polyprotein is processed by viral and cellular proteinases, yielding three structural proteins (E, PrM and C) and seven nonstructural proteins (NS1, NS2a, NS2b, NS3, NS4a, NS4b and NS5). In contrast, the segmented RNA viruses of the *Jingmenvirus* group (such as ALSV and JMTV) host four genomic segments, with a total genome size similar to those of the flaviviruses. Segments 1 and 3 encode proteins related to *Flavivirus* NS5 (containing RNA-dependent RNA polymerase and methyltransferase motifs) and the NS2b–NS3 complex (containing proteinase and RNA helicase motifs), whereas segments 2 and 4 encode the proteins VP1–VP3 that are unrelated to *Flavivirus* proteins and are of unknown origin (Qin *et al.*, 2014 ▸; Wang *et al.*, 2019 ▸). These findings suggest an unusual evolutionary link between the unsegmented and segmented viruses in the *Flaviviridae* family.

The *Flavivirus* NS3 protein is one of the most studied non­structural proteins because of its central role in virus replication. It is the core component of the membrane-bound *Flavivirus* replication complex and has multiple enzymatic activities. NS3 contains an N-terminal protease domain and a C-terminal RNA helicase domain. While the proteinase domain participates in polyprotein processing, the RNA helicase domain is involved in viral RNA capping and synthesis (Ferron *et al.*, 2005 ▸). A collection of NS3 helicase structures from unsegmented flaviviruses have been reported to date, including those from Dengue virus (DENV; Xu *et al.*, 2005 ▸), Yellow fever virus (YFV; Wu *et al.*, 2005 ▸), West Nile virus (WNV; Mastrangelo *et al.*, 2007 ▸), Hepatitis C virus (HCV; Kim *et al.*, 1998 ▸; Cho *et al.*, 1998 ▸) and Zika virus (ZIKV; Jain *et al.*, 2016 ▸; Bukrejewska *et al.*, 2017 ▸; Cao *et al.*, 2016 ▸; Fang *et al.*, 2019 ▸; Li *et al.*, 2018 ▸; Tian, Ji, Yang, Xie *et al.*, 2016 ▸; Tian, Ji, Yang, Zhang *et al.*, 2016 ▸; Xu *et al.*, 2019 ▸; Yang *et al.*, 2018 ▸). These studies demonstrate that NS3 helicases not only exhibit a common fold, but also share a similar mechanism underlying ATP hydrolysis, RNA recognition and unwinding. The putative RNA helicases identified in the NS3-like proteins of the segmented RNA viruses share limited sequence identity with that of *Flavivirus* NS3, raising an intriguing question as to whether the structure and function of the NS3-like helicase is preserved in segmented RNA viruses.

## 2. Materials and methods   

### 2.1. Protein expression and purification   

The DNA encoding the C-terminal helicase domain of ALSV NS3 (NS3-Hel; residues 322–810; GenBank AXE71876.1) was synthesized and inserted into a pET-28a-SUMO vector between the BamHI and XhoI sites, expressing ALSV NS3-Hel with an N-terminal 6×His-SUMO tag. The resulting vector was transformed into *Escherichia coli* BL21(DE3) competent cells. A single colony was picked and the bacterial culture was grown in LB medium containing 50 mg l^−1^ kanamycin at 37°C. Expression was induced by adding 0.5 m*M* isopropyl β-d-1-thiogalactopyranoside (IPTG) when the OD_600_ reached 1.0. The bacterial culture was rapidly cooled to 18°C and shaking was continued at 18°C overnight. The cell pellets were harvested and resuspended in lysis buffer consisting of 50 m*M* Tris–HCl pH 8.0, 150 m*M* NaCl, 10 m*M* imidazole, 1 m*M* phenylmethylsulfonyl fluoride (PMSF), 1 m*M* β-mercaptoethanol. The bacterial cells were lysed by ultrasonication on ice and the cell debris was removed by centrifugation at 20 000 rev min^−1^ for 30 min. The supernatant was filtered through a 0.45 µm filter and then loaded onto Ni–NTA resin pre-equilibrated with lysis buffer. The resin was washed twice with ten column volumes of wash buffer consisting of 50 m*M* Tris–HCl pH 8.0, 100 m*M* NaCl, 20 m*M* imidazole, 1 m*M* PMSF, 1 m*M* β-mercaptoethanol to remove non­specifically bound proteins. Subsequently, the 6×His-SUMO tag was cleaved on the column by adding Ulp1 peptidase at 4°C overnight. The flowthrough containing nontagged ALSV NS3-Hel was collected and subjected to a HiTrap Q HP column (GE Healthcare) pre-equilibrated with buffer consisting of 20 m*M* Tris–HCl pH 8.0, 75 m*M* NaCl. Nontagged ALSV NS3-Hel was eluted with a linear gradient of NaCl from 75 m*M* to 1 *M*.

ALSV NS3-Hel containing l-selenomethionine residues was prepared by transforming the vector into *E. coli* B834 (DE3) competent cells. The bacterial cells were grown in LeMaster medium (Molecular Dimensions) supplemented with l-selenomethionine. The purification of this derivative was the same as described above for the native protein.

Vectors encoding ALSV NS3-Hel mutants were constructed by site-directed mutagenesis (KOD -Plus-; Toyobo) following the manufacturer’s instructions.

### 2.2. Crystallization and structure determination   

The ALSV NS3-Hel protein was concentrated to approximately 2 mg ml^−1^ prior to crystallization trials. Crystallization was performed in a hanging-drop vapor-diffusion setup at 20°C. 1 µl protein sample was mixed with 1.2 µl crystallization buffer consisting of 0.16 *M* calcium acetate, 20%(*v*/*v*) PEG 3350, 5 m*M* tris(2-carboxyethyl)phosphine. The crystals were soaked in crystallization buffer containing 10% ethylene glycol and flash-cooled in liquid nitrogen. X-ray diffraction experiments were conducted on the X06DA beamline at the Swiss Light Source, Paul Scherrer Institute, Villigen, Switzerland. Highly redundant data were collected usings X-rays at a wavelength of 0.9791 Å. The crystal diffracted X-rays to 2.9 Å resolution and belonged to space group *P*2_1_2_1_2_1_. The data were processed using *XDS* (Kabsch, 2010 ▸). *SHELXC*/*D*/*E* were used to locate heavy atoms (Se) and to calculate an initial electron-density map. The preliminary atomic model with a single molecule in the asymmetric unit was built using the *CRANK*2 pipeline in the *CCP*4 package (Winn *et al.*, 2011 ▸). The preliminary model was improved by manual model building using *Coot* (Emsley *et al.*, 2010 ▸). The structure was refined using *Phenix* (Liebschner *et al.*, 2019 ▸). All structural figures were prepared using *PyMOL* (Schrödinger).

### 2.3. ATPase activity assay   

The ATPase assay was performed as described previously (Kuo *et al.*, 1996 ▸). Each reaction mixture (50 µl) consisted of 20 m*M* Tris–HCl pH 8.0, 5 m*M* DTT, 10 m*M* MgCl_2_, a trace amount of [γ-^32^P]-ATP/UTP and the indicated amount of cold ATP/UTP (from 20 µm to 1 m*M*). The reaction was initiated by addition of the enzyme (16 n*M*). The mixtures were incubated at 28°C. At different time points, 2 µl of the reaction mixture was transferred to 2 µl quenching buffer (0.2 *M* EDTA) to stop the reaction. The samples were spotted onto a polyethyleneimine cellulose plate and resolved with a running buffer consisting of 0.8 *M* acetic acid, 0.8 *M* LiCl. The results were visualized and analyzed using a Typhoon Trio Variable Mode Imager (GE Healthcare).

## 3. Results and discussion   

**Figure 1:**
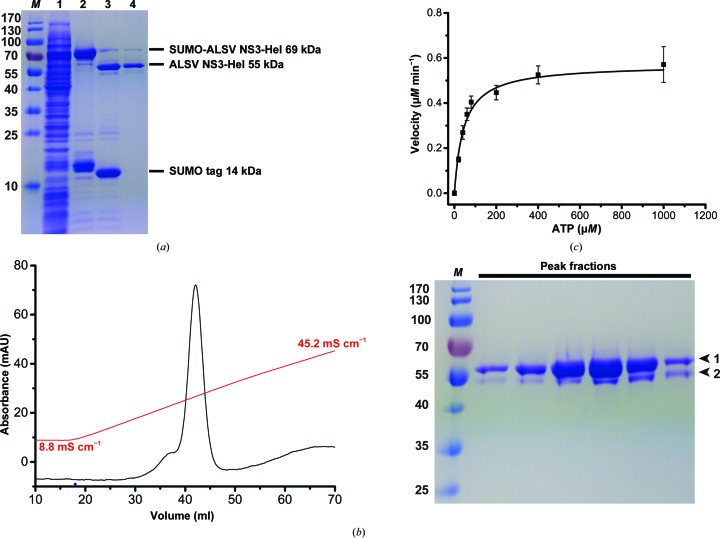
Purification and biochemical characterization of recombinant ALSV NS3-Hel. (*a*) Preliminary purification of ALSV NS3-Hel. Lane 1, supernatant from the bacterial lysis; lane 2, eluate from the Ni–NTA column containing 6×His-SUMO-tagged ALSV NS3-Hel; lane 3, overnight digestion by Ulp1 peptidase; lane 4, flowthrough from the Ni–NTA column, on which the cleaved 6×His-SUMO tag and undigested species were trapped. Lane *M* contains molecular-mass markers (labeled in kDa). (*b*) Left: final purification of ALSV NS3-Hel using ion-exchange chromatography. The nontagged ALSV NS3-Hel was eluted from the HiTrap Q HP column with a linear gradient of NaCl from 75 m*M* to 1 *M*. UV absorbance (280 nm) and the conductivity of the elution buffer are plotted as a function of elution volume. Right: an SDS–PAGE analysis of the eluate fractions corresponding to the UV absorbance peak. Lane *M* contains molecular-mass markers (labeled in kDa). (*c*) ATPase activity of ALSV NS3-Hel. The velocity of ATP hydrolysis is plotted as a function of the ATP concentration. The kinetic parameters *K*_m_ and *V*_max_ were calculated *via* nonlinear fitting to the Michaelis–Menten equation.

To gain structural and functional insights into the putative NS3-like helicase identified in ALSV (GenBank AXE71876.1), we overexpressed the C-terminal portion of ALSV NS3 (residues 322–810) containing the predicted RNA helicase domain, designated ALSV NS3-Hel. A 6×His-SUMO tag was fused to the N-terminus of ALSV NS3-Hel. The recombinant protein was purified and the 6×His-SUMO tag was cleaved using a SUMO-specific proteinase [Figs. 1 ▸(*a*) and 1 ▸(*b*)]. To investigate the enzymatic activity of ALSV NS3-Hel, we performed an ATPase assay. We found that ALSV NS3-Hel could hydrolyze ATP with a *K*_m_ value of 55 ± 8 µ*M* and *k*_cat_ = 0.61 ± 0.04 s^−1^ [Fig. 1 ▸(*c*)]. In addition, we evaluated the UTP hydrolysis activity of ALSV NS3-Hel. The enzyme hydrolyzed UTP with a *K*_m_ value of 185 ± 19 µ*M* and *k*_cat_ = 0.85 ± 0.02 s^−1^ (Supplementary Fig. S1). Hence, the results indicate that ALSV NS3-Hel does not have significant specificity for NTP, which is consistent with the specificities of many other *Flavivirus* NS3 helicases. Finally, we compared the NTP hydrolysis activity of ALSV NS3-Hel with a selection of nonsegmented NS3-Hels (Supplementary Table S1), demonstrating that the NTPase activity of ALSV NS3-Hel is comparable to those measured for characterized nonsegmented NS3-Hels (Tian, Ji, Yang, Zhang *et al.*, 2016 ▸; Suzich *et al.*, 1993 ▸; Warrener *et al.*, 1993 ▸; Jin & Peterson, 1995 ▸; Kuo *et al.*, 1996 ▸; Mancini *et al.*, 2007 ▸; Speroni *et al.*, 2008 ▸; Assenberg *et al.*, 2009 ▸; Yang *et al.*, 2018 ▸; Xu *et al.*, 2019 ▸).

ALSV NS3-Hel shares limited sequence identity (15–28%) with *Flavivirus* NS3 helicases, and none of the available NS3-Hel structures could be used as a homologous model for structure determination. We therefore adopted an *ab initio* phasing strategy. We overexpressed ALSV NS3-Hel in *E. coli* B834 (DE3) cells to obtain a derivative containing selenomethionine residues. The crystals of ALSV NS3-Hel diffracted X-rays to 2.9 Å resolution. The structure was solved using the single-wavelength anomalous dispersion (SAD) method. The final structure has *R*_work_ and *R*_free_ values of 26.1% and 29.2%, respectively. The statistics for data collection, phasing and structure refinement are summarized in Supplementary Tables S2 and S3.

**Figure 2:**
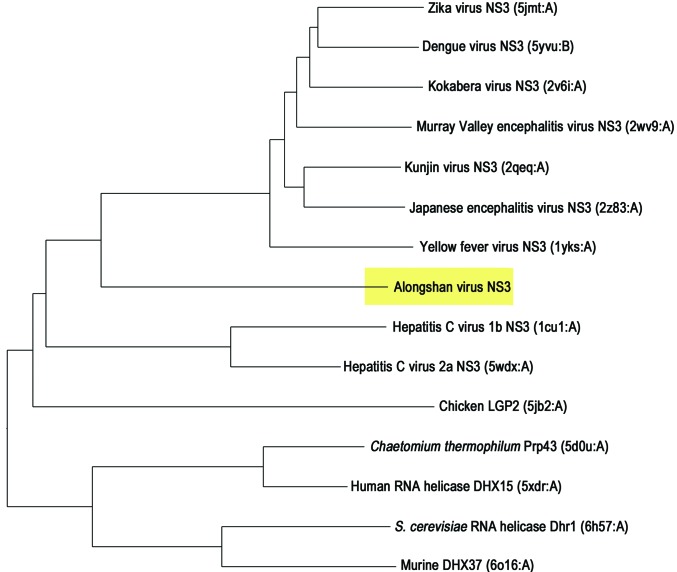
Structure-similarity dendrogram of SF2 helicases structurally related to ALSV NS3-Hel. In a search for structural homologs of ALSV NS3-Hel using the *DALI* server, the most related structures (*DALI* *Z*-­score ≥ 18.0) and the structure of ALSV NS3-Hel were submitted to all-against-all structure comparison (http://ekhidna2.biocenter.helsinki.fi/dali/) to generate the structure-similarity dendrogram. The dendrogram is derived by average linkage clustering of the structure-similarity matrix (*DALI* *Z*-scores). The protein name, PDB code and chain ID are indicated. ALSV NS3 is highlighted with a yellow background.

The overall fold of ALSV NS3-Hel is similar to that of the nonsegmented viral NS3 helicases. We used the *DALI* server (http://ekhidna2.biocenter.helsinki.fi/dali/) to search for structural homologs of ALSV NS3-Hel (Supplementary Table S4) against the PDB90 database. Top hits include a collection of NS3 helicases from various viruses from the *Flaviviridae*, such as Kokobera virus, Japanese encephalitis virus, Murray Valley encephalitis virus, Dengue virus, Zika virus, Kunjin virus, Yellow fever virus and Hepatitis C virus. The *Z*-score ranges from 30.4 to 22.4, while the r.m.s.d. ranges from 3.1 to 4.4 Å. Next, we generated a structure-similarity dendrogram by comparing the most related helicase structures (*DALI* *Z*-score ≥ 18.0; Fig. 2 ▸). A structure-similarity dendrogram was derived by average linkage clustering of the structure-similarity matrix (*DALI* *Z*-scores). The *Z*-scores between ALSV NS3-Hel and *Flavivirus* NS3 helicase structures range from 24.9 to 30.4, with r.m.s.d.s of 2.9–3.2 Å. By contrast, the *Z*-scores between the ALSV NS3-Hel structure and HCV NS3 helicases ranges from 20.7 to 22.3, with r.m.s.d.s of 3.8–4.4 Å. The unusually high r.m.s.d. values may be attributed to the interdomain movement between the D1 and D2 domains triggered by RNA or ATP binding and the less conserved D3 domain. We therefore compared the structure of the isolated domains of ALSV NS3-Hel with their counterparts in various flaviviral NS3 structures (Supplementary Table S4). Superimposing the D1 and D2 domains of ALSV NS3-Hel onto flaviviral NS3 structures gave r.m.s.d.s of 2.0–2.7 and 1.9–2.6 Å, respectively. By contrast, the structure of the D3 domain of ALSV NS3-Hel exhibited very limited similarity to the D3 domain of flaviviral NS3 structures, which is consistent with the high variability of the D3 domain in flaviviral NS3 helicases. These data suggest that ALSV NS3-Hel is structurally more related to *Flavivirus* NS3 helicases.

**Figure 3:**
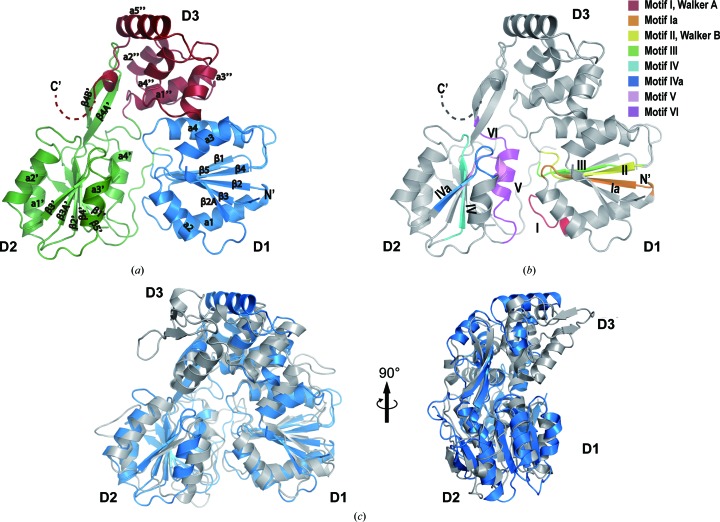
The overall structure of ALSV NS3-Hel. (*a*) Ribbon model of the crystal structure of ALSV NS3-Hel with annotated secondary-structure elements. Three domains, D1, D2 and D3, are indicated. Unless specified otherwise, all illustrations of the ALSV NS-Hel structure in this paper have the same orientation. (*b*) Ribbon model of the ALSV NS3-Hel structure with the conserved motifs highlighted using the indicated color codes. (*c*) Superimposition of the crystal structures of ALSV NS3-Hel and ZIKV NS3-Hel (PDB entry 5jmt; Tian, Ji, Yang, Xie *et al.*, 2016 ▸). Right: the same model rotated around the vertical axis by 90°.

The structure of ALSV NS3-Hel has a flattened triangular shape [Fig. 3 ▸(*a*)], which can be divided into three domains: the N-terminal domains 1 and 2 (D1, residues 322–480; D2, residues 481–640) and the C-terminal domain 3 (D3). D1 and D2 are tandem RecA-like domains with an α/β fold. D1 is composed of six parallel β-sheets (β1, β2, β2A, β3, β4 and β5) sandwiched by four α-helices (α1–α4), whereas D2 contains six parallel β-sheets (β1′, β2′, β3′, β3A′, β4′ and β5′) sandwiched by four α-helices (α1′–α4′). A β-hairpin composed of a pair of antiparallel β-sheets (β4A′–β4B′) protrudes from the D2 domain and interacts with the D3 domain. In particular, the β-hairpin is packed against a hydrophobic patch formed by α1′′, α2′′ and the N-terminus of α5′′ of the D3 domain.

**Figure 4:**
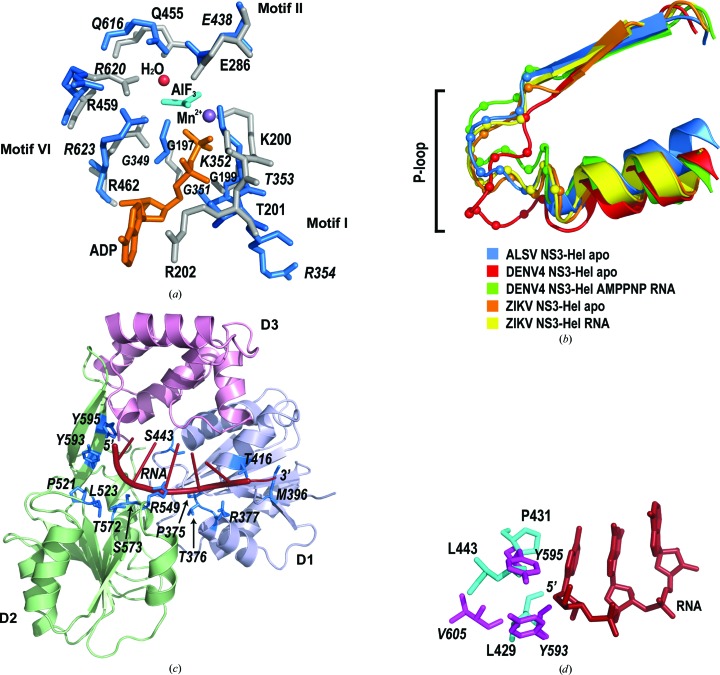
The active site and RNA-binding groove of ALSV NS3-Hel. (*a*) Invariant residues in the active site of ALSV NS3-Hel are shown as stick models (blue); their counterparts in the superimposed ZIKV NS3-Hel (PDB entry 5y6m; Yang *et al.*, 2018 ▸) are shown in gray. The bound ligands ADP (orange), AlF_3_ (cyan) and manganese ion (purple) are also shown. Residues from ALSV NS3-Hel are annotated in italics. (*b*) Structural superimposition of the P-­loops of apo ALSV NS3-Hel (blue), apo DENV4 NS3-Hel (red), DENV4 NS3-Hel complexed with RNA and AMPPNP (green), apo ZIKV NS3-Hel (orange) and ZIKV NS3-Hel complexed with RNA (yellow). (*c*) A model of an ALSV NS3-Hel–RNA complex. The RNA was modeled into the ALSV NS3-Hel structure by superimposition with the DENV4 NS3-Hel–RNA complex (PDB entry 2jlv; Luo *et al.*, 2008 ▸). ALSV NS3-Hel is colored by domain: D1, light blue; D2, light green; D3, pink. Residues that were predicted to contact RNA are colored blue and shown as stick models. (*d*) Aromatic residues located at the 5′ end of the model RNA. Residues from ALSV NS3-Hel are shown as magenta stick models and their structural counterparts in DEVN4 NS3-Hel are shown as cyan stick models. RNA is shown a a red stick model.

Unlike the available NS3-Hel structures [Fig. 3 ▸(*c*)], the D3 domain of ALSV NS3-Hel is partially disordered. While the first half of D3 (α1′′–α5′′; residues 641–735) is visible in the electron-density map, the C-terminal 75 residues (736–810) are missing [Fig. 3 ▸(*a*)]. The last visible residue, Arg735, at the C-terminus of ALSV NS3-Hel extends into the solvent. In the SDS–PAGE analysis of the fractions eluted from a HiTrap Q column [Fig. 1 ▸(*b*)], protein-degradation products were visible. We therefore analyzed the crystals of ALSV NS3-Hel (Supplementary Fig. S2). Multistep transfer of ALSV NS3-Hel crystals to fresh drops of crystallization buffer was carried out to remove free protein. The proteolytic products were present in the crystals and their proportion was clearly increased in comparison with the sample before crystallization. This result indicates that the missing C-terminal portion of the D3 domain might be owing to proteolytic cleavage that occurred during purification despite the presence of a high concentration of proteinase inhibitor (see Section 2). This high susceptibility to proteolysis may reflect an usual intrinsic flexibility of the D3 domain. For example, the D3 domain of the DENV NS3 helicase is implicated in binding the viral RNA-dependent RNA polymerase NS5 (Johansson *et al.*, 2001 ▸). The concave surface between the D2 and D3 domains of the DENV NS3 helicase has been proposed to bind the duplex portion of the RNA substrate ahead of the partially opened fork (Sampath *et al.*, 2006 ▸). Therefore, it is possible that the D3 domain of ALSV NS3-Hel is also involved in the binding of other proteins or RNA, which is essential for the stabilization of its conformation. All conserved SF2 helicase motifs are located in the cleft formed between the D1 and D2 domains. While the D1 domain contains motifs I (P-loop/Walker A), Ia, II (Walker B) and III, the D2 domain contains motifs IV, IVa, V and VI [Fig. 3 ▸(*b*)]. Comparison with the structures of the active site of ZIKV NS3 bound by ATP, ADP–AlF_3_–Mn^2+^ and ADP–Mn^2+^ allowed the identification of all catalytically important residues [Fig. 4 ▸(*a*), Supplementary Fig. S4(*a*) and Supplementary Table S5]. We found that the residues involved in the recognition of NTP, hydrolysis intermediates, catalytic water and metal ion are invariant in ALSV NS3-Hel, which suggests that the catalytic mechanism underlying ATP hydrolysis is conserved in the NS3-Hels from segmented viruses. In motif I, the structural equivalents of Gly197 and Gly199 in ZIKV NS3 are Gly349 and Gly351 in ALSV NS3, suggesting their role in the recognition of the triphosphate moiety of NTP. Thr201 and Glu286 of ZIKV NS3 coordinate an Mn^2+^ ion for NTP hydrolysis, and their equivalents in ALSV NS3 are Thr353 (motif I) and Glu438 (motif II). Arg202 of ZIKV NS3 stabilizes the adenosine base of ADP, and its match in ALSV NS3 is Arg354 (motif I). Lys200, Arg459 and Arg462 of ZIKV NS3 interact with the γ-phosphate of NTP, and their analogs in ALSV NS3 are Lys352, Arg620 and Arg623, respectively. Gln455 of ZIKV NS3 coordinates a catalytic water and the nearby γ-phosphate (or its mimic AlF_3_), and its match in ALSV NS3 is Gln616. While most of the catalytic residues of ALSV NS3-Hel superimpose well with their equivalents in ZIKV NS3, the P-loop of ALSV NS3-Hel adopts a different conformation. In particular, Thr353 and Arg354 of ALSV NS3-Hel do not superimpose with their counterparts in ZIKV NS3 [Fig. 4 ▸(*a*)]. This might be owing to the fact that the structure of ALSV NS3 is in the apo form and ATP binding might induce conformational changes of the P-loop. This speculation is consistent with the finding that the P-loop adopts various conformations in different flaviviral NS3 structures (Yang *et al.*, 2018 ▸), suggesting intrinsic flexibility of this region.

DENV NS3-Hel undergoes major conformational changes upon RNA binding (Luo *et al.*, 2008 ▸). In particular, RNA binding triggers a conformational switch of the P-loop to a catalytically competent state, offering a mechanistic explanation for RNA-stimulated ATP hydrolysis. We superimposed the structure of ALSV NS3-Hel with the structures of DENV4 NS3-Hel and ZIKV NS3-Hel in apo and RNA-bound forms, and compared the structures of the P-loop [Fig. 4 ▸(*b*)]. While the conformational differences of the P-loop of ZIKV NS3-Hel between apo and RNA-bound forms are small, significant conformational changes were observed between the different forms of DENV NS3-Hel. The P-loop conformation of apo ALSV NS3-Hel is more similar to the catalytically competent state than to the unusual P-loop in apo DENV NS3-Hel. A full understanding of the RNA-induced conformational changes of ALSV NS3-Hel will requires the further investigation of enzyme–RNA complexes.

It has been reported that single-stranded RNA binding affects the coordination of the divalent ion, which may affect the catalytic activity and facilitate the release of ADP–Mn^2+^ (Luo *et al.*, 2008 ▸). Thr200 and Glu285 coordinating the divalent ion in DENV NS3-Hel are invariant in ALSV NS3-Hel as Thr353 (motif I) and Glu438 (motif II). We superimposed the ALSV NS3-Hel structure with those of the DENV4 NS3-Hel–AMPPNP–RNA and the DENV4 NS3–AMPPNP complexes (Supplementary Fig. S5). The comparison reveals that the conformation of Glu438 in ALSV NS3-Hel is similar to the conformation of Glu285 of the DENV4 NS3-Hel–AMPPNP–RNA complex, which is not an optimal conformation for ion coordination. Additionally, Thr353 of ALSV NS3-Hel is located ∼4.4 Å apart from its counterpart in DENV4 NS3-Hel. To understand whether ATP or RNA binding triggers the optimal P-loop conformation for divalent ion coordination requires further structure determination of ALSV NS3-Hel in complex with ATP, divalent ion and RNA.

To investigate the interaction between ALSV NS3-Hel and RNA, we modeled a single-stranded RNA into the ALSV NS3-Hel structure by superimposing it with the structure of the DENV4 NS3–RNA–AMPPNP complex (PDB entry 2jlv; Luo *et al.*, 2008 ▸). This analysis revealed a set of residues in ALSV-Hel that may participate in RNA recognition, most of which are highly conserved [Fig. 4 ▸(*c*), Supplementary Fig. S4(*b*) and Supplementary Table S6]. This model demonstrates that the single-stranded RNA is accommodated in the groove separating the D1 and D2 domains from the D3 domain of ALSV NS3-Hel. The 3′ portion of the modeled RNA strand is located on top of the D1 domain. Thr376 and Arg377 (motif Ia) may recognize the phosphodiester backbone of the RNA, whereas Pro375 and Thr416 may recognize the 2′-OH group and Ser443 may recognize the nucleotide base. The 5′ portion of the modeled RNA makes contacts with residues from the D2 domain (motifs IV, IVa and V). Leu523, Arg549 and Thr572 may interact with the RNA backbone, whereas Pro521 and Ser573 may interact with the 2′-OH group of the RNA and Tyr593 and Tyr595 may interact with the RNA base. The β-hairpin extending from the D2 domain (β4A′–β4B′) forms a wall of the RNA-binding tunnel which accommodates the 5′ end of the single-stranded RNA. It is worth noting that the structural counterparts of the two aromatic residues Tyr593 and Tyr595 in ALSV NS3 are the hydrophilic residues Pro431 and Leu429 in DENV4 NS3. Both of these residues are involved in inter­action with the RNA base. The availability of Tyr593 and Tyr595 at this location suggest that they could recognize the RNA base via π–π stacking (Fig. 4*d*). Our multiple sequence alignment indicates that this feature is conserved in the segmented flaviviruses but is not present in the nonsegmented flaviviruses (Supplementary Fig. S3). While the structural counterparts of Tyr593 in the non­segmented flaviviruses are hydrophobic residues, the counterparts of Tyr595 are mostly prolines. Hence, it is possible that the segmented viruses adopt a distinctive mechanism of RNA recognition.

In summary, we provide biochemical and structural characterizations of the NS3-like helicase of ALSV, a member of the segmented virus group in the *Flaviviridae*. Our findings demonstrate that ALSV NS3-Hel exhibits an ATPase activity similar to those of NS3 helicases encoded by unsegmented flaviviruses. ALSV NS3-Hel exhibits an overall fold resembling that of *Flavivirus* NS3 helicases. Structure-similarity analysis showed that ALSV NS3-Hel is more structurally related to *Flavivirus* NS3 helicases than to HCV NS3 helicases. These results reveal the unusual evolutionary link between the unsegmented and segmented RNA viruses of the *Flaviviridae* family from structural and biochemical perspectives. Our results provide a structural framework for the design of antiviral agents and suggest the possibility of developing wide-spectrum antivirals targeting the *Flavivirus* and *Jingmenvirus* groups owing to the shared structural features in their NS3-Hels.

## Supplementary Material

PDB reference: NS3-like helicase from Alongshan virus, 6m40

Supplementary Figures and Tables. DOI: 10.1107/S2052252520003632/jt5043sup1.pdf

## References

[bb1] Assenberg, R., Mastrangelo, E., Walter, T. S., Verma, A., Milani, M., Owens, R. J., Stuart, D. I., Grimes, J. M. & Mancini, E. J. (2009). *J. Virol.* **83**, 12895–12906. 10.1128/JVI.00942-09 PMC2786852 19793813

[bb2] Bukrejewska, M., Derewenda, U., Radwanska, M., Engel, D. A. & Derewenda, Z. S. (2017). *Acta Cryst.* D**73**, 767–774. 10.1107/S2059798317010737 28876240

[bb3] Cao, X., Li, Y., Jin, X., Li, Y., Guo, F. & Jin, T. (2016). *Nucleic Acids Res.* **44**, 10505–10514. 10.1093/nar/gkw941 PMC5137455 27915293

[bb4] Cho, H.-S., Ha, N.-C., Kang, L.-W., Chung, K. M., Back, S. H., Jang, S. K. & Oh, B.-H. (1998). *J. Biol. Chem.* **273**, 15045–15052. 10.1074/jbc.273.24.15045 9614113

[bb6] Emsley, P., Lohkamp, B., Scott, W. G. & Cowtan, K. (2010). *Acta Cryst.* D**66**, 486–501. 10.1107/S0907444910007493 PMC2852313 20383002

[bb7] Fang, J., Jing, X., Lu, G., Xu, Y. & Gong, P. (2019). *ACS Infect. Dis.* **5**, 177–183. 10.1021/acsinfecdis.8b00214 30672289

[bb8] Ferron, F., Rancurel, C., Longhi, S., Cambillau, C., Henrissat, B. & Canard, B. (2005). *J. Gen. Virol.* **86**, 743–749. 10.1099/vir.0.80590-0 15722535

[bb9] Jain, R., Coloma, J., García-Sastre, A. & Aggarwal, A. K. (2016). *Nat. Struct. Mol. Biol.* **23**, 752–754. 10.1038/nsmb.3258 PMC5085289 27399257

[bb10] Jia, N., Liu, H.-B., Ni, X. B., Bell-Sakyi, L., Zheng, Y.-C., Song, J.-L., Li, J., Jiang, B.-G., Wang, Q., Sun, Y., Wei, R., Yuan, T.-T., Xia, L.-Y., Chu, Y.-L., Wei, W., Li, L.-F., Ye, J.-L., Lv, Q. Y., Cui, X.-M., Guan, Y., Tong, Y.-G., Jiang, J.-F., Lam, T. T.-Y. & Cao, W.-C. (2019). *EBioMedicine*, **43**, 317–324. 10.1016/j.ebiom.2019.04.004 PMC6557783 31003930

[bb11] Jin, L. & Peterson, D. L. (1995). *Arch. Biochem. Biophys.* **323**, 47–53. 10.1006/abbi.1995.0008 7487072

[bb12] Johansson, M., Brooks, A. J., Jans, D. A. & Vasudevan, S. G. (2001). *J. Gen. Virol.* **82**, 735–745. 10.1099/0022-1317-82-4-735 11257177

[bb13] Kabsch, W. (2010). *Acta Cryst.* D**66**, 125–132. 10.1107/S0907444909047337 PMC2815665 20124692

[bb14] Kim, J. L., Morgenstern, K. A., Griffith, J. P., Dwyer, M. D., Thomson, J. A., Murcko, M. A., Lin, C. & Caron, P. R. (1998). *Structure*, **6**, 89–100. 10.1016/s0969-2126(98)00010-0 9493270

[bb15] Kuivanen, S., Levanov, L., Kareinen, L., Sironen, T., Jaaskelainen, A. J., Plyusnin, I., Zakham, F., Emmerich, P., Schmidt-Chanasit, J., Hepojoki, J., Smura, T. & Vapalahti, O. (2019). *Euro Surveill.* **24**, 1900394. 10.2807/1560-7917.ES.2019.24.27.1900394 PMC6628756 31290392

[bb16] Kuo, M.-D., Chin, C., Hsu, S.-L., Shiao, J.-Y., Wang, T.-M. & Lin, J.-H. (1996). *J. Gen. Virol.* **77**, 2077–2084. 10.1099/0022-1317-77-9-2077 8811006

[bb17] Li, L., Wang, J., Jia, Z. & Shaw, N. (2018). *Acta Cryst.* F**74**, 205–213. 10.1107/S2053230X18003813 PMC5894106 29633968

[bb5] Liebschner, D., Afonine, P. V., Baker, M. L., Bunkóczi, G., Chen, V. B., Croll, T. I., Hintze, B., Hung, L.-W., Jain, S., McCoy, A. J., Moriarty, N. W., Oeffner, R. D., Poon, B. K., Prisant, M. G., Read, R. J., Richardson, J. S., Richardson, D. C., Sammito, M. D., Sobolev, O. V., Stockwell, D. H., Terwilliger, T. C., Urzhumtsev, A. G., Videau, L. L., Williams, C. J. & Adams, P. D. (2019). *Acta Cryst.* D**75**, 861–877. 10.1107/S2059798319011471 PMC6778852 31588918

[bb18] Luo, D., Xu, T., Watson, R. P., Scherer-Becker, D., Sampath, A., Jahnke, W., Yeong, S. S., Wang, C. H., Lim, S. P., Strongin, A., Vasudevan, S. G. & Lescar, J. (2008). *EMBO J.* **27**, 3209–3219. 10.1038/emboj.2008.232 PMC2599875 19008861

[bb19] Mancini, E. J., Assenberg, R., Verma, A., Walter, T. S., Tuma, R., Grimes, J. M., Owens, R. J. & Stuart, D. I. (2007). *Protein Sci.* **16**, 2294–2300. 10.1110/ps.072843107 PMC2204129 17893366

[bb20] Mastrangelo, E., Milani, M., Bollati, M., Selisko, B., Peyrane, F., Pandini, V., Sorrentino, G., Canard, B., Konarev, P. V., Svergun, D. I., de Lamballerie, X., Coutard, B., Khromykh, A. A. & Bolognesi, M. (2007). *J. Mol. Biol.* **372**, 444–455. 10.1016/j.jmb.2007.06.055 17658551

[bb21] Qin, X.-C., Shi, M., Tian, J.-H., Lin, X.-D., Gao, D.-Y., He, J.-R., Wang, J.-B., Li, C.-X., Kang, Y.-J., Yu, B., Zhou, D.-J., Xu, J., Plyusnin, A., Holmes, E. C. & Zhang, Y.-Z. (2014). *Proc. Natl Acad. Sci. USA*, **111**, 6744–6749. 10.1073/pnas.1324194111 PMC4020047 24753611

[bb22] Sampath, A., Xu, T., Chao, A., Luo, D., Lescar, J. & Vasudevan, S. G. (2006). *J. Virol.* **80**, 6686–6690. 10.1128/JVI.02215-05 PMC1488930 16775356

[bb23] Speroni, S., Decolibus, L., Mastrangelo, E., Gould, E., Coutard, B., Forrester, N. L., Blanc, S., Canard, B. & Mattevi, A. (2008). *Proteins*, **70**, 1120–1123. 10.1002/prot.21812 18004778

[bb24] Suzich, J. A., Tamura, J. K., Palmer-Hill, F., Warrener, P., Grakoui, A., Rice, C. M., Feinstone, S. M. & Collett, M. S. (1993). *J. Virol.* **67**, 6152–6158. 10.1128/jvi.67.10.6152-6158.1993 PMC238037 8396675

[bb25] Tian, H., Ji, X., Yang, X., Xie, W., Yang, K., Chen, C., Wu, C., Chi, H., Mu, Z., Wang, Z. & Yang, H. (2016). *Protein Cell*, **7**, 450–454. 10.1007/s13238-016-0275-4 PMC4887331 27172988

[bb26] Tian, H., Ji, X., Yang, X., Zhang, Z., Lu, Z., Yang, K., Chen, C., Zhao, Q., Chi, H., Mu, Z., Xie, W., Wang, Z., Lou, H., Yang, H. & Rao, Z. (2016). *Protein Cell*, **7**, 562–570. 10.1007/s13238-016-0293-2 PMC4980333 27430951

[bb27] Wang, Z.-D., Wang, B., Wei, F., Han, S.-Z., Zhang, L., Yang, Z.-T., Yan, Y., Lv, X.-L., Li, L., Wang, S.-C., Song, M.-X., Zhang, H.-J., Huang, S.-J., Chen, J., Huang, F.-Q., Li, S., Liu, H.-H., Hong, J., Jin, Y.-L., Wang, W., Zhou, J.-Y. & Liu, Q. (2019). *N. Engl. J. Med.* **380**, 2116–2125. 10.1056/NEJMoa1805068 31141633

[bb28] Warrener, P., Tamura, J. K. & Collett, M. S. (1993). *J. Virol.* **67**, 989–996. 10.1128/jvi.67.2.989-996.1993 PMC237453 8380474

[bb40] Winn, M. D., Ballard, C. C., Cowtan, K. D., Dodson, E. J., Emsley, P., Evans, P. R., Keegan, R. M., Krissinel, E. B., Leslie, A. G. W., McCoy, A., McNicholas, S. J., Murshudov, G. N., Pannu, N. S., Potterton, E. A., Powell, H. R., Read, R. J., Vagin, A. & Wilson, K. S. (2011). *Acta Cryst.* D**67**, 235–242. 10.1107/S0907444910045749 PMC3069738 21460441

[bb29] Wu, J., Bera, A. K., Kuhn, R. J. & Smith, J. L. (2005). *J. Virol.* **79**, 10268–10277. 10.1128/JVI.79.16.10268-10277.2005 PMC1182653 16051820

[bb30] Xu, S., Ci, Y., Wang, L., Yang, Y., Zhang, L., Xu, C., Qin, C. & Shi, L. (2019). *Nucleic Acids Res.* **47**, 8693–8707. 10.1093/nar/gkz650 PMC6895266 31361901

[bb31] Xu, T., Sampath, A., Chao, A., Wen, D., Nanao, M., Chene, P., Vasudevan, S. G. & Lescar, J. (2005). *J. Virol.* **79**, 10278–10288. 10.1128/JVI.79.16.10278-10288.2005 PMC1182654 16051821

[bb32] Yang, X., Chen, C., Tian, H., Chi, H., Mu, Z., Zhang, T., Yang, K., Zhao, Q., Liu, X., Wang, Z., Ji, X. & Yang, H. (2018). *FASEB J.* **32**, 5250–5257. 10.1096/fj.201701140R 29913559

