# Supplementary material for: Crystal structure of the NS3-like helicase from Alongshan virus
Source: IUCrJ. 2020 Apr 10;7(Pt 3):375–82. doi: 10.1107/S2052252520003632 (PMC7201283; doi:10.1107/S2052252520003632)
Supplement: Supplementary file 1 [file m-07-00375-sup1.pdf]

IUCrJ

Volume 7 (2020)

Supporting information for article:

Crystal structure of the NS3-like helicase from Alongshan virus

Xiaopan Gao, Kaixiang Zhu, Justyna Aleksandra Wojdyla, Pu Chen, Bo Qin, Ziheng Li, Meitian Wang and Sheng Cui

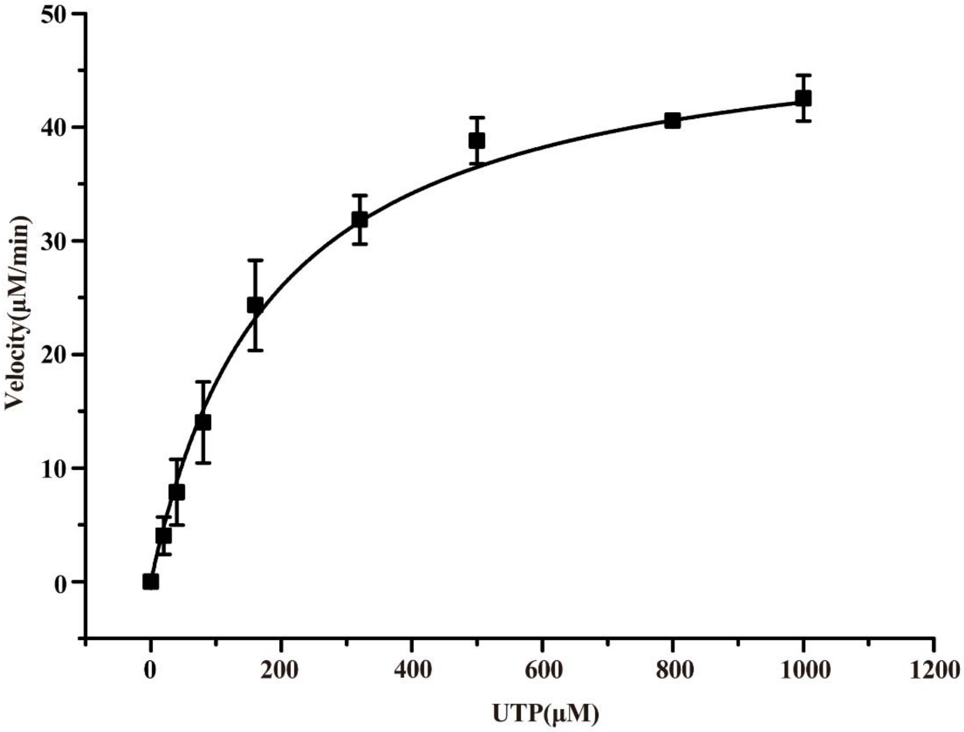

Figure S1 UTP hydrolysis activity of ALSV-NS3-Hel. The velocity of UTP hydrolysis is plotted as the function of the concentration of UTP. The data was fitted using the Michaelis-Menten equation. Enzyme (ALSV NS3-Hel) concentration was 980nM.

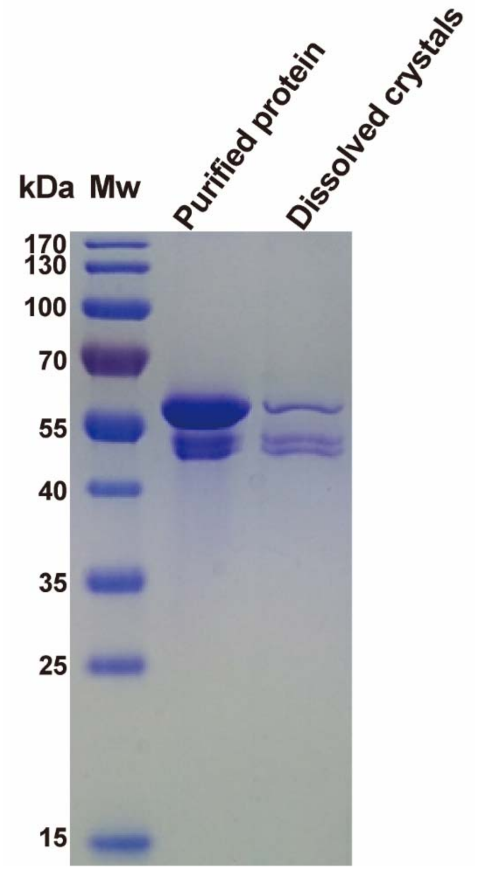

Figure S2 SDS-PAGE analysis of ALSV NS3-Hel crystals. SDS-PAGE analysis of the finally purified ALSV NS3-Hel protein sample before crystallization trials and the crystals of ALSV NS3-Hel. Multiple crystals were transferred to fresh drops of crystallization buffer. The transfer continued from the first drop to the third drop to ensure the complete removal free proteins. The crystals were finally dissolved in SDS loading buffer, heat denatured and analyzed by SDS-PAGE.

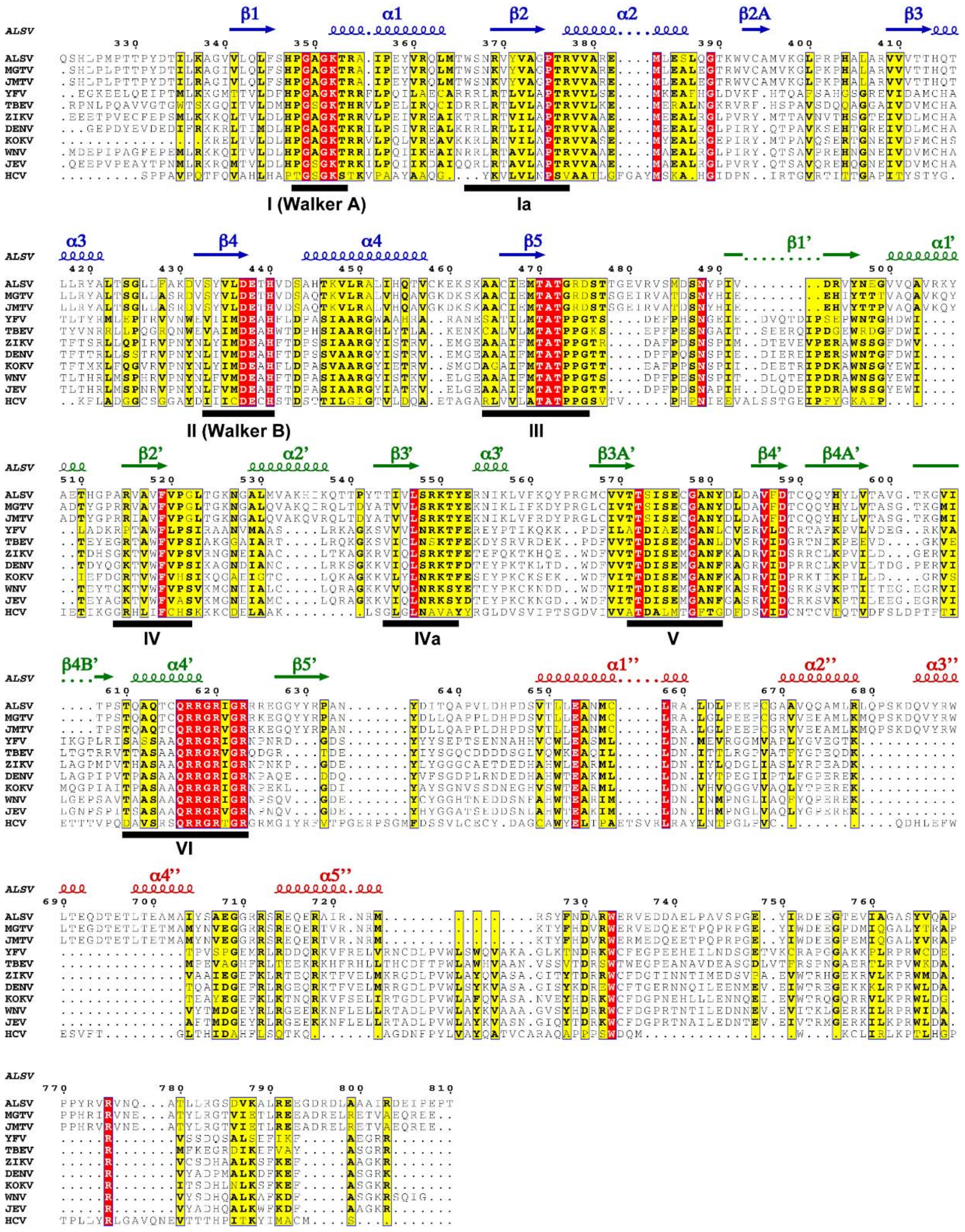

Figure S3 Structure-based multiple sequence alignment of NS3 RNA helicases from unsegmented and segmented viruses. Amino acid sequences NS3 helicases from Yellow fever viurs (YFV), Tick-borne encephalitis virus (TBEV), Zika virus(ZIKV), Dengue virus(DENV), Kokobera virus (KOKV),West Nile virus(WNV), Japanese encephalitis virus (JEV) and Hepatitis C virus (HCV) were aligned with the sequence of the NS3-like helicases from the segmented viruses Mogiana tick virus (MGTV), Jingmen tick virus(JMTV) and Alongshan virus(ALSV) using by the software MUSCLE. Secondary structure elements of

ALSV NS3-Hel are indicated on top of the sequencess using the ESPript server. Residues in with the red background indicate invariant reisdues; residues with the yellow backgroud indicates the conserved residues. Conserved helicase motifs are indicted at the bottom of the sequences.

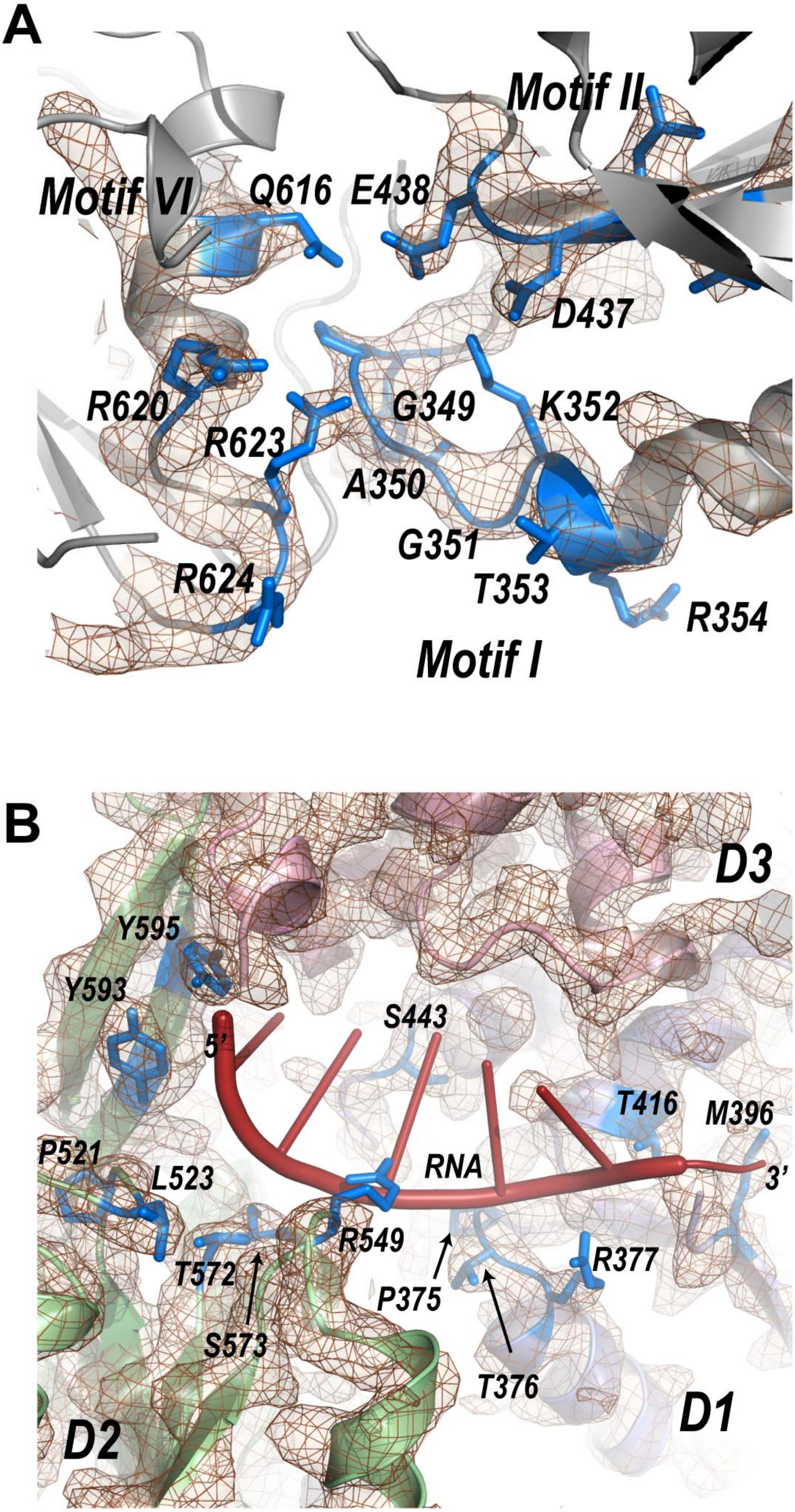
A
Motif II
Motif VI
Q616
E438
D437
R620
G349
K352
R623
A350
G351
R624
T353
R354
Motif I
B
D3
Y595
Y593
S443
5'
T416
M396
P521
RNA
3'
L523
R549
R377
T572
P375
S573
T376
D1
D2

Figure S4 Electron-density map of ATPase active site and putative RNA binding groove of ALSV NS3-Hel. (A) The active site of ALSV NS3-Hel shown with ribbon model and superimposed final 2Fo-Fc electron density map, contour 1.3 s. Invariant residues from Motif I, II and VI are shown in stick model, colored in blue. (B) Magnified view of the model of ALSV NS3-Hel-RNA complex with superimposed final 2Fo-Fc electron density map, contour 1.3 s. The color scheme is the same as in Figure 4C. Residues that were predicted to contact RNA are shown with the stick models

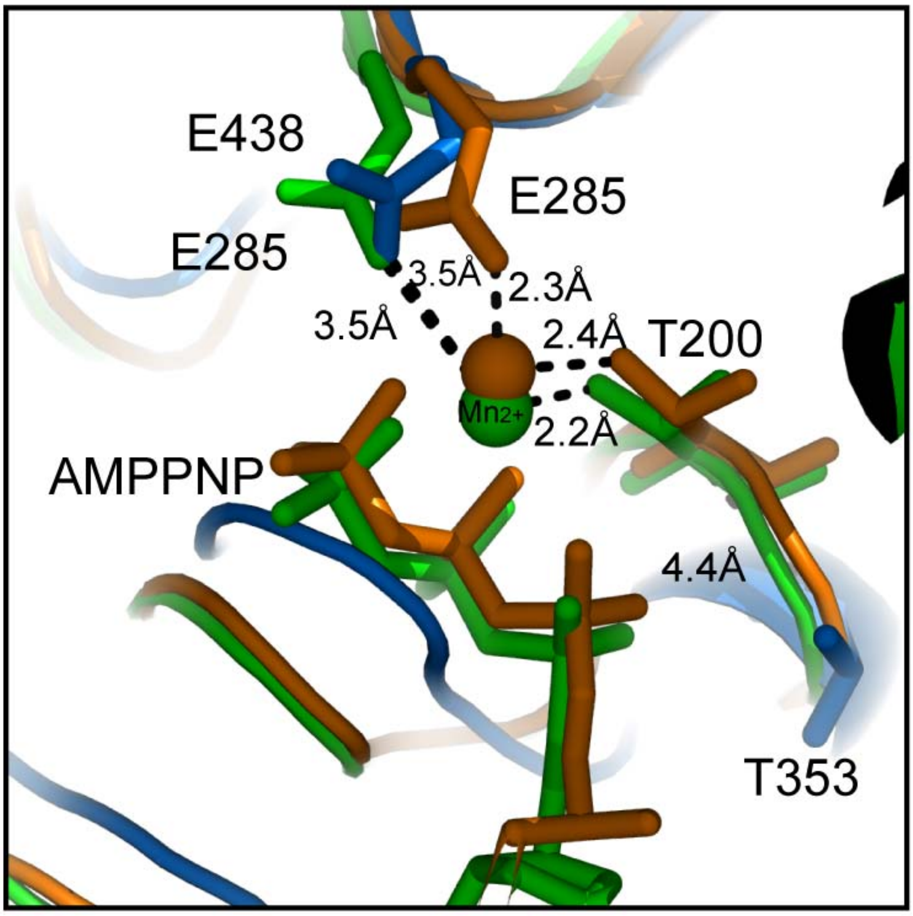

Figure S5 Structural comparison of divalent ion coordination. The structure of ALSV NS3-Hel (blue) was superimposed with DENV NS3-Hel-AMPPNP-RNA complex (PDB ID:2JLV, green) and DENV NS3-AMPPNP complex (PDB ID:2JLR, orange). Residues participating divalent ion coordination are shown with the stick models.

Table S1 The enzymatic activities the NS3 helicase from various Flaviviridae virus

Virus	**Substrate**				**References**
	**ATP**		**UTP**		
	K_m	K_{cat}	K_m	K_{cat}	
Alongshan Virus (ALSV)	55.3±7.7μM	0.61±0.04 s^{-1}	185.43±18.94μM	0.85±0.02 s^{-1}	This paper
Dengue virus (DENV)	34±3μM	6.9s^{-1}			(Xu *et al.*, 2005)
Kokobera virus (KOKV)	340μM	3.7 s^{-1}			(Speroni *et al.*, 2008)
Murray valley encephalitis virus (MVEV)	190±30μM	5.5 s^{-1}			(Assenberg *et al.*, 2009)
	380±30μM	5.3 s^{-1}			(Mancini *et al.*, 2007)
Zika virus(ZIKV)	191±26μM	2.3±0.1 s^{-1}			(Tian *et al.*, 2016)
	89±11.2μM	1.18±0.05s^{-1}			(Yang *et al.*, 2018)
Yellow fever viurs (YFV)	210μM	2.9 s^{-1}	190μM	2.5 s^{-1}	(Warrener *et al.*, 1993)
Japanese encephalitis virus (JEV)	180μM	8.1 s^{-1}			(Kuo *et al.*, 1996)
Hepatitis C virus (HCV)	59μM	3 s^{-1}	100μM	2 s^{-1}	(Suzich *et al.*, 1993)

	90±10μM	6.7±0.8s^{-1}			(Jin & Peterson, 1995)

Table S2 Data collection and refinement statistics

	**ALSV NS3-Hel Se-Met crystal (PDB ID: 6M40)**
**Data collection**	
Space group	$P2_12_12_1$
Cell dimensions	
a, b, c (Å)	42.88, 58.68, 191.47
α, β, γ (°)	90.00, 90.00, 90.00
X ray source	
Wavelength (Å)	0.979144
Data range (Å)	47.87-2.89
Reflections unique	20593 [a]
R_{sym} [b] (last shell)	0.179 (1.44)
$I / \sigma I$ (last shell)	9.68 (1.59)
CC(1/2)	99.6 (74.9)
Completeness (%) (last shell)	99.1 (96.4)
Redundancy (last shell)	7.05 (6.79)
**Refinement**	
Resolution range (Å)	47.87-2.89
Reflections, cut-off, % reflections in cross validation	20578 [a], 1.33, 4.54
R_{work} [c]/ R_{free} [d] (last shell)	0.2463/0.2939 (0.3329/0.4049)
**Atoms**	
Non-hydrogen protein atoms	2993
Protein	2993

Solvent	0
B-factors average (Å^2)	86.10
Protein (Å^2)	86.10
Ligands (Å^2)	
Solvent (Å^2)	
**r.m.s.d**	
Bond lengths (Å)	0.006
Bond angles (°)	0.819
% residues in favored regions, allowed regions, outliers in Ramachandran plot	91.91, 7.28, 0.81

[a] Friedel pairs are treated as different reflections

[b] $R_{sym} = \sum_{hkl}\sum_j |I_{hkl,j} - I_{hkl}|/\sum_{hkl}\sum_j I_{hkl,j}$, where I_{hkl} is the average of symmetry-related observations of a unique reflection

[c] $R_{work} = \sum_{hkl} ||F_{obs}(hkl)|-|F_{calc}(hkl)||/\sum_{hkl}|F_{obs}(hkl)|$.

[d] R_{free}= the cross-validation R factor for 5% of reflections against which the model was not refined.

Table S3 Phasing Statistics of ALSV NS3-Hel crystal containing Se-Met.

**Compound**	Se
**Number of sites**	7
**Figure-of-merit acentric/centric**	0.294/0.138
**Phasing power acentric**	0.957
**R-Cullis for acentric**	0.835

Table S4 Dali search of ALSV-NS3-Hel structure against PDB90 database[a] (top 10 Z-score hits).

**No.**	**Chain[b]**	**Z[c]**	**Rmsd（D1, D2, D3）**	**lali[d]**	**nres[e]**	**%id[f]**	**Description**
**1:**	2v6i-A	30.9	3.0 (2.0, 2.0, 3.9)	334	421	22	MOLECULE: RNA HELICASE;
**2:**	2Z83-A	28.9	3.3 (2.2, 2.0, N.D.)	334	426	24	MOLECULE: HELICASE/NUCLEOSIDE TRIPHOSPHATASE;
**3:**	2wv9 - A	28.7	3.2 (2.1, 1.9, N.D.)	336	600	23	MOLECULE:FLAVIVIRIN PROTEASE NS2B REGULATORY SUBUNIT, FLAV;
**4:**	5yvu- B	28.5	3.2 (2.1 2.1, 3.7)	335	601	24	MOLECULE:

							GENOME POLYPROTEIN;
**5:**	2bmf -B	28.2	3.1 (2.0, 2.0, N.D.)	334	443	28	MOLECULE: NS3 HELICASE;
**6:**	5jmt -A	27.8	3.2 (2.1, 2.3, N.D.)	333	443	23	MOLECULE: RNA HELICASE;
**7:**	2qeq -A	27.3	3.1 (2.1, 2.0, 3.8)	327	415	25	MOLECULE:FL AVIVIRIN PROTEASE NS3 CATALYTIC SUBUNIT;
**8:**	1yks -A	26.8	3.3 (2.1, 2.5, 4.0)	330	431	21	MOLECULE: GENOME POLYPROTEIN [CONTAINS: FLAVIVIRIN;
**9:**	2qeq -B	25.0	3.1 (2.0, 1.9, 4.0)	311	391	23	MOLECULE: FLAVIVIRIN PROTEASE NS3 CATALYTIC SUBUNIT;
**10:**	5wdx - A	22.8	4.5 (2.7, 2.6, N.D.)	322	642	15	MOLECULE: JFH-1 NS3;

[a] PDB90 is a non-redundant subset of Protein Data Bank structures with less than 90% sequence identity to each other.

[b] PDB code and chain ID

[c] Dali Z-score

[d] The number of aligned C-alpha atoms

[e] The number of C-alpha atoms in the database structure

[f] Amino acids sequence identity

N.D. Not determined.

Table S5 NS3 NTPase active site comparison between ZIKV and ALSV

Motifs	ALSV	ZIKV
Phosphates Motif I	G349<br>G351	G197<br>G199
R-finger, motif VI	R623	R462
Base stabilization	R354	R202
Ribose recognition	N488	N330
Metal ion coordination	E438<br>T353	E286<br>T201
Gamma phosphate (AlF3) recognition	K352<br>E438<br>R620<br>R623<br>Q616	K200<br>E286<br>R459<br>R462<br>Q455

Table S6 . Prediction of ALSV NS3-Hel residues participating in RNA recognition.

ALSV NS3-Hel	*Dengue 4 NS3-Hel (PDB: 2JLV)*	*Location*	*RNA recognition*
*Y593	P431	D2	base
*Y595	L429	D2	
*S443	D290	D1	
P521	P363	D2	sugar 2’-OH
*S573	D409	D2	
P375	P223	D1	
T416	T264	D1	
*M396	Q243	D1	
L523	I365	D2	phosphate backbone
R549	R387	D2	
T572	T408	D2	
R377	R225	D1	
T376	T224	D1	

* residues not conserved in ALSV NS3-Hel.

References

Assenberg, R., Mastrangelo, E., Walter, T. S., Verma, A., Milani, M., Owens, R. J., Stuart, D. I., Grimes, J. M. & Mancini, E. J. (2009). *Journal of virology* **83**, 12895-12906.

Jin, L. & Peterson, D. L. (1995). *Archives of biochemistry and biophysics* **323**, 47-53.

Kuo, M. D., Chin, C., Hsu, S. L., Shiao, J. Y., Wang, T. M. & Lin, J. H. (1996). *The Journal of general virology* **77 (Pt 9)**, 2077-2084.

Mancini, E. J., Assenberg, R., Verma, A., Walter, T. S., Tuma, R., Grimes, J. M., Owens, R. J. & Stuart, D. I. (2007). *Protein science : a publication of the Protein Society* **16**, 2294-2300.

Speroni, S., De Colibus, L., Mastrangelo, E., Gould, E., Coutard, B., Forrester, N. L., Blanc, S., Canard, B. & Mattevi, A. (2008). *Proteins* **70**, 1120-1123.

Suzich, J. A., Tamura, J. K., Palmer-Hill, F., Warrener, P., Grakoui, A., Rice, C. M., Feinstone, S. M. & Collett, M. S. (1993). *Journal of virology* **67**, 6152-6158.

Tian, H., Ji, X., Yang, X., Zhang, Z., Lu, Z., Yang, K., Chen, C., Zhao, Q., Chi, H., Mu, Z., Xie, W., Wang, Z., Lou, H., Yang, H. & Rao, Z. (2016). *Protein & cell* **7**, 562-570.

Warrener, P., Tamura, J. K. & Collett, M. S. (1993). *Journal of virology* **67**, 989-996.

Xu, T., Sampath, A., Chao, A., Wen, D., Nanao, M., Chene, P., Vasudevan, S. G. & Lescar, J. (2005). *J Virol* **79**, 10278-10288.

Yang, X., Chen, C., Tian, H., Chi, H., Mu, Z., Zhang, T., Yang, K., Zhao, Q., Liu, X., Wang, Z., Ji, X. & Yang, H. (2018). *FASEB J* **32**, 5250-5257.
